# Morbid Obesity Due to Prolactinoma and Significant Weight Loss After Dopamine Agonist Treatment

**DOI:** 10.1016/j.aace.2021.01.004

**Published:** 2021-03-11

**Authors:** Muzaffar Ali, Lubna Mirza

**Affiliations:** 1 Department of Internal Medicine, Rehman Medical Institute, Peshawar, Pakistan; 2 Department of Endocrinology, Norman Regional Hospital, Oklahoma

**Keywords:** dopamine agonist, endocrinology, morbid obesity, prolactinoma, weight loss, DA, dopamine agonists, FSH, follicle-stimulating hormone, LH, luteinizing hormone

## Abstract

**Objective:**

Morbid obesity may be related to a prolactinoma, although uncommon, and can lead to adverse effects like insulin resistance and metabolic syndrome. Recent research suggests that hyperprolactinemia causes an abnormal lipid profile, weight gain, and cardiovascular diseases. Moreover, high prolactin levels lead to decreased testosterone production by disrupting 17-b-estradiol synthesis. Our objective was to present a case of prolactinoma with morbid obesity, hypogonadism, and then significant weight loss after dopamine agonist treatment.

**Methods:**

The clinical course, in addition to serial laboratory and imaging results, are presented. These include prolactin levels, testosterone levels, thyroid function tests, blood sugar levels, and serial lipid profiles.

**Results:**

In this report, we discuss a case of 30-year-old male with prolactin-secreting macroadenoma with clinical features of hypogonadism, hypothyroidism, and morbid obesity. He showed marked improvement in obesity and hypogonadism with dopamine agonist therapy supplemented with clomiphene citrate.

**Conclusion:**

Prolactinomas with morbid obesity can be successfully treated contingent upon proper medication and compliance with medications. Insulin resistance, hypogonadism, prolactin levels, body mass index, and tumor size all improved by regular follow-up and treatment adherence.

## Introduction

Apart from the observed effects of prolactin on lactation and gonadal function, it is also known to have significant clinical implications on metabolism.^1^ Recently, there has been a lot of research linking prolactinoma with insulin resistance and metabolic syndrome.^2^^,^^3^

The likely pathogenesis of weight gain in hyperprolactinemia includes: (i) decreased dopaminergic tone^4^; (ii) leptin resistance^5^; (iii) reduction in adiponectin levels^6^; (iv) high hypothalamic pressure^7^; and (v) hypogonadism.^8^ Studies suggest that high prolactin can cause a shift in glucose homeostasis and insulin sensitivity. It can also lead to increased low-density lipoproteins and triglycerides and reduced high-density lipoproteins levels, which is likely the result of reduced lipoprotein lipase activity.^9^ This can lead to further weight gain and increased risk of cardiovascular diseases. Dopamine agonists (DA) are used to regulate prolactin levels, body weight, glucose metabolism, and lipid homeostasis.^10^

Hyperprolactinemia leads to weight gain and infertility in both genders. In men, it leads to erectile dysfunction, loss of libido, low testosterone levels, reduced ejaculate volume, and oligospermia.^11^

High prolactin levels can cause decreased testosterone production by disrupting 17-b-estradiol synthesis from Leydig cells, which has adverse effects on spermatogenesis.^12^^,^^13^

Here, we discuss the case of a 30-year-old male presenting with features of hypogonadism and resistant obesity. He did not visit the nutritionist nor exercise on a regular basis. He was found to have a prolactin-secreting macroadenoma and showed clinical improvement with dopamine agonist therapy, that is, cabergoline and clomiphene citrate.

## Case Report

A 30-year-old male was referred to the endocrinology department with high thyroid stimulating hormone, low testosterone, and low follicle-stimulating hormone (FSH)/luteinizing hormone (LH) levels. He had previously seen a psychiatrist for depression, decreased concentration, tiredness, lack of motivation, loss of libido, negative mood, and insomnia. He had gained a significant amount of weight apparently due to the lack of physical activity. After his baseline investigations, he was found to have hypothyroidism and low testosterone levels. The psychiatrist started the patient on oral levothyroxine 25 mcg daily, but there was no improvement in his clinical condition. So, the psychiatrist referred the patient to endocrinology services.

Further history revealed fatigue, hoarseness of voice, tinnitus, anxiety, weakness, excessive sweating, and intermittent blurred vision. His past medical history was positive for depression and anxiety. His medications included oral sertraline HCl 50 mg once daily. There was no history of galactorrhea, hearing loss, gynecomastia, head trauma, seizure disorder, or loss of consciousness.

On physical examination, the patient was morbidly obese (body mass index = 45.67 kg/m^2^) with normal vital signs. The thyroid examination was normal. The genitourinary assessment showed Tanner Stage 3, and urethral meatus was normal. Furthermore, bilateral testicles were small and measured 10 mL in size. He had scanty pubic hair, a micro-penis, and showed incomplete secondary sexual characteristics. The rest of the systemic examination was unremarkable.

Laboratory investigations showed hyperprolactinemia (315 ng/mL). The pituitary workup showed secondary/central hypogonadism and secondary hypothyroidism. Anti-thyroid peroxidase antibodies were done to rule out autoimmune thyroiditis and were negative. Thyroid stimulating hormone was 4.28, free thyroxine 0.86, total cholesterol 153, high-density lipoproteins 45, low-density lipoproteins 91, and thyroglobulin 87. Magnetic resonance imaging of the head revealed 17 mm pituitary macroadenoma pressing on the optic chiasm.

The patient was ultimately diagnosed with a prolactin-secreting pituitary macroadenoma and was started on cabergoline 0.25 mg twice weekly. The dose was increased to 0.5 mg twice weekly, along with levothyroxine 100 mcg daily. He was not prescribed testosterone replacement therapy at that time as the physician decided to monitor him for endogenous testosterone production with the improvement in prolactin levels.

After 3 months of treatment, the prolactin levels dropped to 71 ng/mL, with improvement in vision and headache. Follow-up after 6 months revealed a further reduction in prolactin levels (32 ng/mL). For the low testosterone and low FSH/LH levels, he was started on clomiphene citrate 50 mg twice weekly in January of 2018. At the same visit, he was prescribed phentermine and topiramate 3.75-23 mg once daily for his obesity but did not take this drug due to expense. He was referred to the dietitian for complete nutritional assessment and guidance, but did not show up despite multiple attempts on behalf of the nutritionist to contact him.

On subsequent follow-up, he showed improvement in prolactin levels from 32 ng/mL to 5 ng/mL, total/free testosterone was in the high normal range (total testosterone: 791 ng/dL, free testosterone: 140.7 pg/mL), thyroid function tests were normal, and there was a marked reduction in body mass index with a weight loss of more than 100 pounds.

## Discussion

DA therapy (cabergoline) can improve clinical and laboratory parameters in patients with prolactinoma. In addition to starting our patient on cabergoline, we also gave him levothyroxine. This led to an improvement in his thyroid profile and testosterone levels. Our approach is consistent with current literature, where similar regimens were used in patients presenting with prolactinoma, leading to hypogonadism and hypothyroidism. In other cases, an initial dose of 0.25 mg of cabergoline twice weekly was sufficient to exert significant clinical response, that is, the patient showed improvement in vision as early as day 3 of cabergoline therapy (see Table). Along with the DA treatment, patients with prolactinoma can also be given 100 mcg of levothyroxine and 250 mg testosterone injections every 3 weeks with notable improvement in their thyroid profile and gonadal function.^14^ We did not need to start testosterone injections because he responded to clomiphene.

**Table:** Study Parameters Before and After Cabergoline Treatment

	Nov 2016 (initial)	March 2017	April 2020	Reference range
BMI (kg/m^2^)	45.67	42.58	33.77	17.5-25
Weight (lbs)	336.8	314	249	--
Height (inches)	72	72	72	--
Blood pressure	132/74	116/66	140/68	80/120
FSH (IU//L)	1.4	1.6	4.1	1.5-12.4
LH (IU//L)	1.12	1.24	3.9	1.24-7.8
TSH (mIU/L)	7	1.13	3.89	0.5-5
Free thyroxine (T4) (ng/dL)	0.84	1.1	1.21	0.9-2.3
Total testosterone (ng/dL)	110	240	791	280-1100
Free testosterone	0.1%	0.3%	2.1%	1.6-3.3%

Abbreviations: BMI = body mass index; FSH = follicle-stimulating hormone; LH = luteinizing hormone; TSH = thyroid stimulating hormone.

Moreover, DA therapy can cause weight loss by improving insulin and leptin sensitivity and the lipid profile.^15^ In our case, the patient showed a similar response, where he lost a significant amount of weight in addition to the correction in his prolactin levels, thyroid profile, and gonadal function, which was augmented by clomiphene citrate.

The testosterone levels in our patient were low due to central hypogonadism. He was not initially started on testosterone treatment and was monitored for endogenous testosterone production following DA and clomiphene citrate. Our approach is supported by current literature, where secondary hypogonadism due to prolactinoma is not initially treated with testosterone replacement therapy, as testosterone levels might improve with DA therapy,^16^ but after multiple adjustments in the DA dose, clomiphene was added in June of 2017 because the FSH/LH and testosterone levels remained low.

Furthermore, erectile dysfunction and loss of libido are clinical indications for the use of clomiphene citrate, which increases FSH and LH in hypogonadal men, thus increasing testosterone levels. According to the International Society for the Study of Aging Male, values of testosterone below 200 ng/mL are an indication for initiating testosterone therapy. Patients with values of testosterone between 200 and 400 ng/mL can be given a clinical trial of testosterone medications.^17^ Because our patient did not show significant improvement in gonadal function with DA therapy and the patient fulfilled the International Society for the Study of Aging Male criteria for the initiation of testosterone therapy, we started the patient on clomiphene citrate, which led to an improvement in testosterone levels.

## Conclusion

In summary, our case showed overall improvement in prolactin levels, central hypogonadism, and weight reduction after DA treatment and clomiphene citrate. There were no reported side effects, and the efficacy of treatment was evident by the reversal of symptoms such as improvement in vision, drastic weight reduction, and normalization of thyroid and gonadal functions. A follow-up magnetic resonance imaging was not performed due to cost and the fact that symptoms had improved.

Observations from a single case cannot establish causality. Prospective studies will be needed to establish any effects of DA therapy on body weight and adiposity.

## Disclosures

The authors have no multiplicity of interest to disclose.
